# A Behavioral Evaluation of Sex Differences in a Mouse Model of Severe Neuronal Migration Disorder

**DOI:** 10.1371/journal.pone.0073144

**Published:** 2013-09-09

**Authors:** Dongnhu T. Truong, Ashley Bonet, Amanda R. Rendall, Glenn D. Rosen, Roslyn H. Fitch

**Affiliations:** 1 Department of Psychology/Behavioral Neuroscience, University of Connecticut, Storrs, Connecticut, United States of America; 2 Department of Neurology, Beth Israel Deaconess Medical Center, Boston, Massachusetts, United States of America; Université Pierre et Marie Curie, France

## Abstract

Disruption of neuronal migration in humans is associated with a wide range of behavioral and cognitive outcomes including severe intellectual disability, language impairment, and social dysfunction. Furthermore, malformations of cortical development have been observed in a number of neurodevelopmental disorders (e.g. autism and dyslexia), where boys are much more commonly diagnosed than girls (estimates around 4 to 1). The use of rodent models provides an excellent means to examine how sex may modulate behavioral outcomes in the presence of comparable abnormal neuroanatomical presentations. Initially characterized by Rosen et al. 2012, the BXD29- *Tlr4^lps−2J^*/J mouse mutant exhibits a highly penetrant neuroanatomical phenotype that consists of bilateral midline subcortical nodular heterotopia with partial callosal agenesis. In the current study, we confirm our initial findings of a severe impairment in rapid auditory processing in affected male mice. We also report that BXD29- *Tlr4^lps−2J^*/J (mutant) female mice show no sparing of rapid auditory processing, and in fact show deficits similar to mutant males. Interestingly, female BXD29- *Tlr4^lps−2J^*/J mice *do* display superiority in Morris water maze performance as compared to wild type females, an affect not seen in mutant males. Finally, we report new evidence that BXD29- *Tlr4^lps−2J^*/J mice, in general, show evidence of hyper-social behaviors. In closing, the use of the BXD29- *Tlr4^lps−2J^*/J strain of mice – with its strong behavioral and neuroanatomical phenotype – may be highly useful in characterizing sex independent versus dependent mechanisms that interact with neural reorganization, as well as clinically relevant abnormal behavior resulting from aberrant neuronal migration.

## Introduction

Disorders of neuronal migration show substantial heterogeneity in both neuroanatomical and behavioral outcomes, and have been linked to a wide range of neurological, neurobehavioral, and psychiatric disorders including epilepsy, schizophrenia, intellectual disability, autism, and dyslexia [1]–[5]. Associated neuropathology can include neural ectopias and dysplasias, polymicrogyria, and periventricular nodular heterotopia, all of which have been shown to be modulated by both genetic and/or environmental mechanisms (see [6] and [7] for review). These types of focal neuronal migration anomalies can lead to further disorganization of the developing cortex, including abnormal cortical layering and altered patterns of neuronal connectivity [8], [9].

Given the range of neuropathologies associated with neuronal migration disorders, it is not surprising that the severity and types of behavioral and cognitive outcomes also vary greatly depending upon the location, degree, and extent of cortical disruption (see [7] for review). For example, in humans periventricular nodular heterotopia, polymicrogyria, and neural ectopias are generally seen to be localized to the perisylvian region – a well studied area implicated in language processing. Accordingly, these types of anomalies tend to be associated with language and reading disorders in humans [3], [5], [10], [11]. More diffuse neuronal migration anomalies such as classic lissencephaly and subcortical band heterotopia are more commonly associated with severe intellectual disabilities and motor impairments [2], [12], [13]. However, there still remains a general multiplicity of behavioral, cognitive, and neurological outcomes that may result from a neuronal migration disorder, thus making overall patient outcomes difficult to clinically predict exclusively from an anatomical profile.

With regards to elucidating the relationship between different types of neuronal migration anomalies and behavioral outcomes, the use of rodent models has provided an invaluable tool. For example, rodent studies have examined disruption of normal cortical development through injury (focal freeze lesion; [14]–[17]), embryonic exposure to teratogens [18], and/or genetic factors (either intrinsic or genetically manipulated; [19]–[21]). Results show that differing types of neuropathology can lead to different types of anomalous behavioral outcomes. For example, research examining rodent models of focally disrupted neuronal migration (similar to those observed in clinical populations with language and reading disorders) found different combinations of behavioral impairments associated with specific patterns of cortical disruption [14], [15], [18]–[26].

Within the clinical literature, additional evidence reveals that males are more commonly diagnosed with neurodevelopmental disorders, including those associated with language learning impairments, and are also at a greater behavioral disadvantage than females when diagnosed with the same disorder (see [27], for review). However, the mechanisms underlying these sex differences in incidence and severity of behavioral symptomatology remain unclear. Rodent models in the past have directly examined sex differences in both neuroanatomical and behavioral outcome of injury-induced and spontaneously occurring models of disrupted neuronal migration [23], [24]. These studies revealed sex differences in rapid auditory processing (RAP) ability – a behavior associated with and used to model fundamental aspects of language-related ability. Specifically, male subjects with cortical malformations were impaired in short duration auditory processing conditions in comparison to age-matched male controls (no cortical malformation), while female subjects with similar induced/spontaneous cortical malformations performed comparably to their age-matched female controls [23], [24]. Additionally, a study conducted by Rial et al. 2009, described impairments in short term social recognition memory in male mice with focally induced microgyria, while females with the same induced malformation showed no deficit [28]. Together, these studies suggested that females with cortical malformations were not behaviorally impaired on acoustic RAP tasks, as well as a short term social recognition memory task, while comparable malformations in male subjects did lead to deleterious behavioral performance. Despite this experimental evidence, the clinical literature clearly includes reports of cognitive, language, and motor impairments in some females with neuronal migration anomalies – indicating that behavioral impairments can arise from migrational anomalies in females under *at least some circumstances* [7].

In a study investigating the genetic modulation of neuronal migration in hundreds of strains of BXD recombinant inbred (RI) mice, it was found that a particular strain – BXD29/TyJ –showed bilateral midline nodular hetereotopia and partial callosal agenesis in mice born from 2004 onward. Those mice born prior to 1998 were unaffected [29]. Interestingly, earlier research, discovered that genetic variation had occurred in this same strain that made them insensitive to lipopolysaccharide, a bacterial endotoxin [30], [31]. Subsequent study revealed a repeated sequence added to both ends of the *Tlr4* gene. On the basis of these findings, BXD29/TyJ RI mice were re-derived from a 1979 cryopreserved embryonic stock and designated BXD29/Ty (wildtype for *Tlr4*), while the RI strain with the *Tlr4* spontaneous mutation was redesignated as BXD29-*Tlr4* ^lps−2J^/J. Rosen and colleagues reported that the malformation observed in BXD29-*Tlr4* ^lps−2J^/J mice was 100% penetrant in both males and females, and the location of neuroanatomical anomaly was invariant (between retrosplenial cortex and somatosensory/visual cortices) [29]. Moreover, the malformation was found to develop in later stages of cortical migration [29]. In addition, specific and severe impairment in RAP was found in male mice, but with no concurrent behavioral deficits in spatial or nonspatial maze learning (Morris and nonspatial water maze, respectively), or sensorimotor ability (rotarod; [29]). Concurrent examination of the coisogenic BXD29/Ty (wildtype) mouse strain, revealed no aberrant neuroanatomical or behavioral profile [29]. However, genetic backcross experiments indicated that the neuroanatomical phenotype was most likely not mediated by the *Tlr4* spontaneous mutation indentified by Cook et al. [31]. Instead, results suggested at least two autosomal recessive genes contributed to the observed phenotype [29].

The current study was performed to provide a more comprehensive examination of the relationship between severe neuroanatomical malformation and behaviors clinically associated with neuronal migration disorders, including language-related and social dysfunctions, by expanding the neurobehavioral profile of the BXD29- *Tlr4^lps−2J^*/J recombinant inbred strain of mouse. Additionally, an investigation of potential sex differences was undertaken to determine whether BXD29- *Tlr4^lps−2J^*/J females display the same pattern of behavioral outcomes as their male BXD29- *Tlr4^lps−2J^*/J counterparts. Based on prior data, we hypothesized that affected females might show no deficits [23], [24]. In this regard, examination of sex differences could provide insight into whether some form of developmental or behavioral compensation or “protection” in female subjects is evident in this particular model of highly disrupted neuronal migration.

We report here that we replicated our previous findings of a severe RAP impairment in male BXD29- *Tlr4^lps−2J^*/J mutant mice [29]. Interestingly, concurrent examination of RAP in female BXD29- *Tlr4^lps−2J^*/J mutant mice revealed no mutation by sex interaction, meaning that female BXD29- *Tlr4^lps−2J^*/J mice performed comparably to BXD29- *Tlr4^lps−2J^*/J males and were equally impaired relative to control females. However, further examination of Morris water maze learning did reveal sex differences in performance, with female BXD29- *Tlr4^lps−2J^*/J exhibiting *superior* Morris water maze ability compared to both male BXD29- *Tlr4^lps−2J^*/J mutant mice and female coisogenic controls. Finally, novel examination of social behavior and vocalizations revealed that both male and female BXD29- *Tlr4^lps−2J^*/J mutant mice displayed deviant social behaviors in comparison to BXD29/Ty wildtype controls (though vocalizations could only be measured in males since adult females rarely vocalize [32]). These anomalies were unexpectedly found in a *hyper-*social direction.

## Materials and Methods

### Ethics Statement

All procedures were conducted in compliance with the National Institutes of Health and approved by the University of Connecticut's Institutional Animal Care and Use Committee (IACUC; protocol A09-050M).

### Selection of Behavioral Tasks

The selection of behavioral tasks was based on the intent to; 1) replicate and expand the original behavioral findings described in Rosen et al. 2013; 2) examine potential sex differences using these tasks; and 3) to employ new assessments of social behaviors. Tasks examined included auditory processing tasks, rotarod, Morris water maze, male social vocalization behavior, and a social preference task. Our aims included: 1) to provide a more comprehensive neurobehavioral profile of BXD29-*Tlr4^lps−2J^*/J utilizing a behavioral battery reflective of the wide range of cognitive and social behaviors associated with neuronal migration disorders and; 2) to examine whether severe neuroanatomical anomalies could lead to sexually dimorphic behavioral outcomes.

### Subjects

For the series of behavioral experiments, ten male and ten female BXD29-*Tlr4^lps−2J^*/J mutant (JAX stock number 000029) and ten male and ten female BXD29/Ty wildtype (JAX stock number 010981) were obtained from the Jackson Laboratory (Bar Harbor, ME) at postnatal day 29–36. Note that in subsequent text, BXD29-*Tlr4^lps−2J^*/J will be referred to as “mutant” and BXD29/Ty will be referred to as “wildtype”. All subjects arrived together at the University of Connecticut, Department of Psychology, and were single-housed in standard lab cages (12 h/12 h light/dark cycle) with food and water available *ad lib*. Subjects were behaviorally examined together in adulthood beginning on postnatal day 129 (P129). All procedures were performed blind to subject genotype.

### Auditory Processing: The Startle Reduction Paradigm

Assessment of RAP was conducted utilizing a modified pre-pulse inhibition paradigm (PPI; see [33], for detailed review). Briefly, the startle reduction paradigm exploits the subject's acoustic startle reflex (ASR) – a large amplitude motor reflex which is evoked by an unexpected, intense, auditory stimulus (startle eliciting stimulus; SES). However, a reduction in the ASR can be elicited with the presentation of a non-startling but salient, stimulus (i.e. pre-pulse or cue) 20–500 ms prior to the SES. If the subject is capable of detecting the auditory pre-pulse preceding the SES, then attenuation of the ASR is typically seen. In this way the PPI paradigm provides a means to examine auditory detection and discrimination using varied manipulations of the pre-pulse cue. Attenuation of the ASR to the SES is quantified and examined using an “attenuated score” (ATT), which is a percent comparison of the ASR amplitude over cued and uncued trials (cued ASR/uncued ASR*100).

During auditory testing, subjects were placed on individual load-cell platforms (MED Associates, St. Albans, VT). Voltage output from each load cell platform was sent through a linear amplifier (PHM-250-60 MED) into a Biopac MP100WS Acquisition system (Biopac Systems, Goleta, CA). The MP100WS was connected to a Macintosh computer running Acqknowledge v 3.9.2, which recorded the ASR of the subject (in volts) for each trial following the presentation of a SES. For data analysis, the maximum peak value of the ASR was extracted from the 200 ms epoch following the onset of the SES. The magnitudes of peak values were coded for each cued and uncued trial (representing the subject's absolute response amplitude for each trial). Auditory stimuli were generated using a Dell Pentium IV PC with custom programs executed using the program RPvdsEx and a Tucker Davis Technologies (Alachua, FL) real time processor (RP2). Sounds were amplified using a Niles SI-1260 Systems Integration Amplifier (Niles Audio Corporation, Carlsbad, CA), and delivered via powered speakers located approximately 50 cm above each platform. For all auditory processing paradigms, the SES was a 50 ms broadband white noise “burst” presented at 105 dB.

### Auditory Processing: Normal Single Tone

Prior to RAP examination, all subjects were assessed on the normal single tone task – a measure of baseline auditory and PPI ability. Specifically, the auditory PPI control task examined whether subjects exhibited hearing deficits and/or impaired gross motor reflexes which could confound further auditory PPI testing. Testing sessions consisted of 104 pseudorandomly presented cued and uncued trials at inter-trial intervals (ITI) of varying durations (16–24 s). The task comprised a silent background with the intermittent presentation of an intense SES (50 ms, 105 dB white noise burst) during uncued trials. However, cued trials were characterized by a salient, yet moderately intense, auditory cue (50 ms, 75 dB, 5000 Hz tone pip) presented 100 ms prior to the SES. A percent comparison of cued and uncued response amplitudes (ASR) using an attenuated score (cued ASR/uncued ASR*100; ATT) were utilized for analysis.

### Auditory Processing: Silent Gap Detection

The silent gap detection task examined the subject's ability to discriminate breaks (silent gaps) in continuous broadband white noise. Daily test sessions comprised 300 pseudorandomly presented cued and uncued trials, which were characterized by a continuous presentation of white noise (75 dB) with the occurrence of the SES at varying ITIs (16–24 s). Uncued trials consisted of a 0 ms gap condition (no cue) prior to the presentation of the SES, and provided the subject's baseline ASR amplitude score relative to cued trials. During cued trials, silent gaps of variable duration were embedded within the background white noise and presented 100 ms prior to SES onset. Two different variations of the silent gap detection paradigm were utilized – a long duration gap and a short duration gap task. The long gap detection task utilizes silent gap durations ranging from 50 to 300 ms (SG 0–300 ms) across three consecutive days of testing, beginning on P132. For the short gap detection task, subjects were assessed using silent gap durations ranging between 2 to 100 ms (SG 0–100 ms) across four consecutive days of testing (beginning on P182). Again, attenuated scores (ATT) derived by a percent comparison of cued and uncued response amplitudes (cued ASR/uncued ASR*100) were assessed.

### Sensorimotor Assessment: Rotarod

The rotarod task was used to examine subject's general sensorimotor abilities and balance. On P156, subjects were individually placed on a rotating cylindrical drum that gradually accelerated from 4 to 40 rotations per minute over a span of two minutes. Subjects were provided four consecutive test trials on the rotarod, and the length of time to remain on the rotating drum was recorded. Latency on the accelerating drum was averaged across the four trials for further analysis.

### Water Maze Assessment: Visual Platform

All subjects were examined on the visual platform control task to rule out any potential underlying differences and/or impairments in motivation, swimming, or visual ability that could impair subject's ability to effectively perform subsequent tasks (and thus exclude them from further water maze assessment). On P157, subjects were placed in one end of an oval tub (103 cm×55.5 cm) filled with room temperature water. Here, they were required to swim to a visible platform (8.5 cm in diameter; 1 cm above water surface), located at the opposite end of the tub. Latency to reach the platform was recorded for analysis.

### Water Maze Assessment: Morris Water Maze

The Morris water maze is a behavioral task that is commonly used to assess spatial learning and memory, and specifically the ability to locate the position of a submerged escape platform using various static extra maze cues. Beginning on P160, subjects were tested on the Morris water maze over a span of four consecutive test days (sessions). During each test session, subjects were given four trials to locate the submerged platform. However, for each test trial, the subject release point into the maze was selected pseudorandomly at one of the four compass locations around the maze (i.e. north, south, east, and west), with each point used once per test session. Subjects were allowed 45 seconds to complete the trial and find the escape platform. If the platform was not located prior to the 45 second allotment, subjects were gently guided to the goal before removal from the maze. The position of the hidden platform remained static throughout all four test sessions. Latency to the escape platform was measured and recorded using a Sony camera integrated with a SMART video tracking program (Panlab, Barcelona, Spain).

### Social Context Assessment: Male Vocalizations

Male mice produce ultrasonic vocalizations when they are in the presence of a female (and particularly during estrus), or can detect a female's urinary estrus pheromones [34]. Unfortunately, female mice do not vocalize substantially (primarily at low levels to other females), and therefore we did not include females for this task [32]. Specifically, we measured the vocalization emission of ten male BXD29/Ty and ten male BXD29-*Tlr4^lps−2J^*/J mice when exposed to accumulated seven-day dirty bedding obtained from mature, age-matched, female BXD29/Ty and BXD29- *Tlr4^lps−2J^*/J mice. Bedding from seven-days was used to ensure inclusion of estrus phase (which is a 4 day cycle; [35]). On P251, male subjects were individually placed in a standard laboratory cage filled with the bedding, and here vocalization behavior was recorded for 120 seconds using a ¼ inch condenser microphone (Brüel & Kjær type 4136, Nærum, Denmark) suspended 10 cm above the test subject. The microphone signal was preamplified with a Brüel & Kjær type 2619 preamplifier and then amplified using a Brüel & Kjær type 2636 amplifier (Brüel & Kjær, Nærum, Denmark). The signal was digitized at a sampling rate of 200 kHz using a Tucker Davis Technologies (Alachua, FL) multifunction processor (RX6) and saved as a.wav file using a custom MATLAB program (MathWorks, Natick, MA) on a Dell Pentium IV PC. Recorded sound waveforms were visualized and examined using Adobe Audition (Adobe, San Jose, CA). Total time spent vocalizing was calculated by extracting vocalization intervals (continuous vocalization epochs<200 ms apart) from periods of silence (no vocalization behavior). Percent time vocalizing for each subject was calculated by dividing the duration of active vocalization by the total time (120 seconds), multiplied by 100.

### Social Context Assessment: Social Preference Task

The social preference task was adapted from a social approach task utilized by Nadler et al. 2004, and further detailed by Crawley, 2004, to examine social interaction behaviors of mice when presented with a stranger mouse [36], [37]. This task was included because in the clinical literature for patients with malformations of cortical development such as neuronal migration anomalies, these anomalies are also associated with aberrant social behaviors [4], [38]–[41]. The purpose of the task was to determine whether mutants displayed differential social preference (sociability) behaviors in comparison to wildtypes when presented simultaneously with a stranger mouse and a novel toy, as well as to examine potential sex differences in social preference associated with neuronal migration anomalies. The social preference apparatus was similar in design to those previously described [36], [37]. Briefly, the test box consisted of a 40.5 cm (length) ×21.5 cm (width) ×19 cm (deep) acrylic box separated into three chambers using two removable partitions. The removable partitions each contained openings that could open and close at the beginning and end of the test session to allow the mouse to freely move between the three chambers. The task was conducted in a darkened room with the lamp situated ∼20 cm above the center chamber. Prior to the initiation of the test trial, subjects were placed in the center chamber for two minutes with the doors of the partitions closed to prevent the subject from moving into the other chambers. This allowed the mouse to habituate to the center chamber. Before the beginning of the test trial, an age and sex-matched wildtype stranger mouse was placed inside a 9 cm×9 cm×9 cm wire cage in one of the flanking chambers, while a novel object (toy rubber band ball) was placed inside a similar wire cage in the opposite flanking chamber. At the end of the habituation trial, the test trial began with the simultaneous opening of the partition doors to allow free movement among all three chambers. Using a stop watch, an observer (blind to genotype) recorded the time intervals spent in each chamber over a span of five minutes. Following the end of the five minutes, the test subject was removed from the test chambers and returned to the home cage. Total time spent in each chamber (stranger mouse, center, and novel object) was measured in seconds, and the percent time the test subject spent with the stranger mouse over the novel object was calculated by subtracting the total time spent with the novel toy from the total time spent with the stranger mouse, divided by the total test session (300 sec), multiplied by 100 [{total time with stranger mouse (sec) – total with novel toy (sec)}/300(sec)]*100.

### Histology

Following behavioral testing, all subjects were weighed, deeply anesthetized with a mixture of ketamine (100 mg/kg) and xylazine (15 mg/kg), and transcardially perfused using a 0.9% saline solution followed by 4% paraformaldehyde. Brains were extracted, bottled in paraformaldehyde, and shipped to GDR at Beth Israel Deaconess Medical Center for further histological preparation. Brains were embedded in 12% celloidin and sliced in the coronal plane at a 30 µm thickness. A 1-in-5 series of sections were mounted and stained for Nissl substance using Thionin. Stereo Investigator System (MBF Biosciences, Williston, VT, USA) integrated with a Zeiss Axio Imager A2 microscope (Carl Zeiss, Thornwood, NY) was utilized to confirm and analyze the presence of malformations in BXD29-*Tlr4^lps−2J^*/J mutants, as well as lack of malformations in BXD29/Ty wildtypes. Estimation of cortical and heterotopia volumes were performed using point counting and Cavalieri's estimator. For statistical analysis, heterotopia volumes were analyzed as percent of neocortical volume (total heterotopia volume/total neocortical volume ×100). Estimation of the number of neurons present in the heterotopia was assessed using the Optical Fractionator probe. A one-way ANOVA was used to determine the presence of Sex differences in BXD29-*Tlr4^lps−2J^*/J mutant mice.

## Results

### Histological confirmation of neuromorphological phenotype – no sex differences in stereological analysis

**Figure 1:**
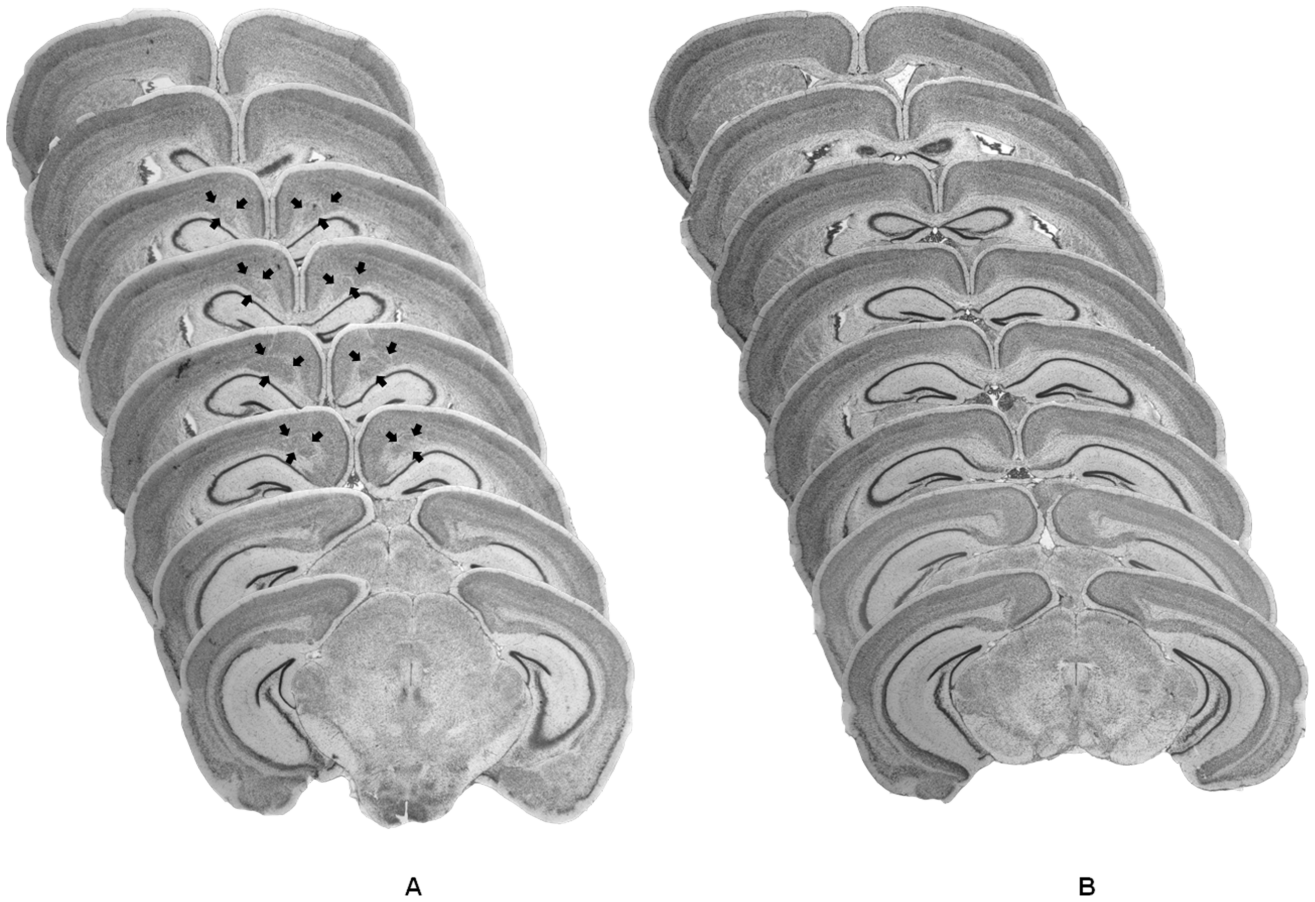
Histological examination of Nissl stained coronal sections in BXD29-*Tlr4^lps−2J^*/J mutant and BXD29/Ty wildtype mice. A) Bilateral midline subcortical nodular heterotopia are present in all BXD29-*Tlr4^lps−2J^*/J mutant mice. Black arrows indicate the boundaries of the abnormal clustering of cells within the coronal plane. B) Visualization of both male and female BXD29/Ty wildtype brains show no evidence of abnormal gross neuromorphology.

Postmortem histological analysis using light microscopy revealed and confirmed the presence of bilateral midline subcortical nodular heterotopia in all (male and female) BXD29-*Tlr4^lps−2J^*/J mutant subjects, as previously reported ([29]; Figure 1A). Visualization of male and female BXD29/Ty wildtype brains did not reveal any gross abnormal neuromophological phenotype, as described elsewhere ([29]; Figure 1B). Analysis of variance revealed no significant Sex differences in heterotopia volume as a percentage of total neocortical volume [F(1,18)  = 1.77, N.S.] in mutant subjects. Mean volume of heterotopia was 2.42% (SEM±0.23) of neocortical volume in female mutant subjects and 2.08% (SEM±0.11) in male mutant subjects. In addition, no Sex differences were observed in the estimated number of neurons present in the heterotopia [F(1,18)  = 2.87, N.S.]. Heterotopias contained an estimated 187,275 (SEM±12,014) neurons in female mutant subjects and 150,444 (SEM±18,132) neurons in male mutant subjects.

### Male and female BXD29-Tlr4^lps−2J^/J mutants show deficits in rapid auditory processing

#### Normal Single Tone

**Figure 2:**
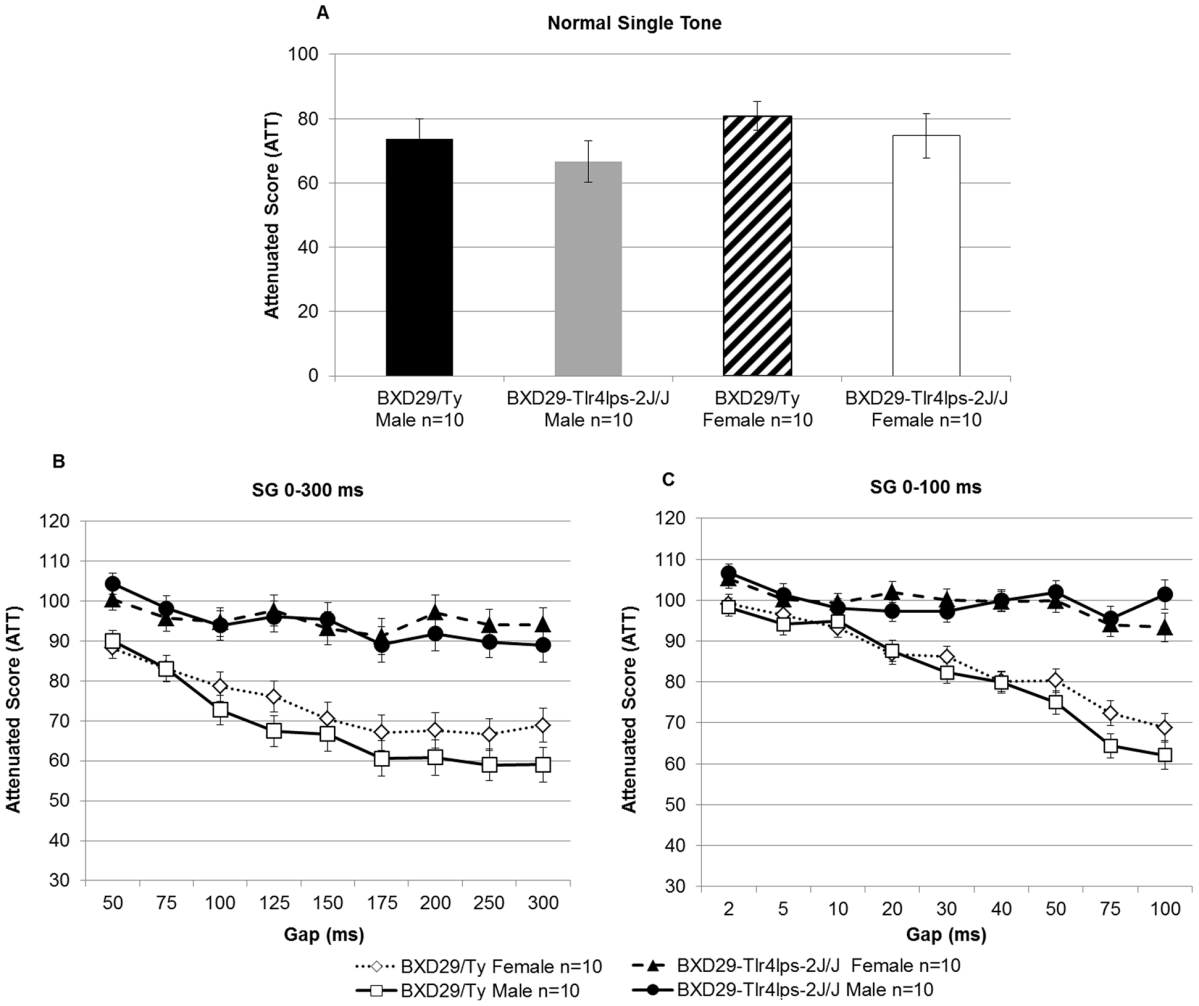
Auditory processing of BXD29-*Tlr4^lps−2J^*/J mutant and BXD29/Ty wildtype mice. A) Assessment of baseline auditory pre-pulse inhibition indicated comparable hearing and pre-pulse inhibition ability across Sex and Strain. Note, a lower attenuation score indicates better auditory pre-pulse inhibition ability. B) Silent Gap 0–300 ms and C) Silent Gap 0–100 ms gap detection tasks showed that BXD29-*Tlr4^lps−2J^*/J mutant mice were specifically impaired in rapid auditory processing. The lack of Sex × Strain interaction signified no sex differences in RAP across both tasks, suggesting that female BXD29-*Tlr4^lps−2J^*/J mutant mice did not exhibit a behavioral sparring of rapid auditory processing ability.

Results from the normal single tone auditory pre-pulse inhibition control task showed that BXD29/Ty males and females, as well as BXD29-*Tlr4^lps−2J^*/J males and females, were all able to detect this simple cue as measured by a comparison of cued versus uncued ASR (*t*(9)  = 3.8, *P*<0.01; *t*(9)  = 2.8, *P*<0.05; *t*(9)  = 4.3, *P*<0.05, *t*(9)  = 3.0, *P*<0.05; respectively, Figure 2A). These results provide evidence that both mutant and wildtype subjects were equally able to hear the auditory cue and had normal pre-pulse inhibition, an interpretation confirmed by a lack of significant main effects of Strain [*F*(1,36)  = 1.2, NS], Sex [*F*(1,36)  = 1.6, NS], or Stain × Sex interaction [*F*(1,36) <1, NS] for the attenuated (ATT) scores.

#### Silent Gap 0–300 ms

Analysis of silent gap ATT scores using a repeated measures ANOVA with between variables of Strain (2 levels: mutant and wildtype) and Sex (2 levels: male and female), and within variables Day (3 levels) and Gap (9 levels), revealed a significant main effect of Strain [*F*(1,36)  = 46.4, *P*<0.001], but no significant main effect of Sex [*F*(1,36) <1, NS], nor a Sex × Strain interaction [*F*(1,36) <1, NS] (Figure 2B). Overall, the results from the silent gap 0–300 ms task show that mutant mice, regardless of sex, performed much worse than wildtype mice. Furthermore, the lack of an interaction between Sex and Strain demonstrates that behavioral effects associated with the mutation are comparable across the sexes. Additionally, a significant interaction between Strain × Gap [*F*(8,288)  = 10.3, *P*<0.001] showed that wildtype mice performed much better on the longer (easier) gap intervals (>125 ms) in comparison to mutant mice, while both groups had more difficulty detecting the shorter gap intervals (<125 ms). An examination of performance at individual gaps using a paired samples t-test comparing mean cued and uncued startle responses in the mutant mice found that they showed significant discrimination for silent gap intervals greater than 75 ms (thus confirming that differences in silent gap 0–300 ms performance between mutant and wildtype mice were a result of poorer RAP ability in mutant mice and *not* an across-the-board failure of gap detection in the mutants). A similar gap detection analysis in wildtype mice found that they were able to discriminate the cue at all gap durations down to 50 ms (as compared to the 75 ms mutant threshold; Figure 2B).

#### Silent Gap 0–100 ms

A repeated measures ANOVA on ATT scores including Strain (2 levels: mutant and wildtype) and Sex (2 levels: male and female) as between variables, and Day (4 levels) and Gap (9 levels) as within variables, revealed a significant main effect of Strain [*F*(1,36)  = 64.9, *P*<0.001]. However, there was no main effect of Sex [*F*(1,36) <1, NS] or Sex × Stain interaction [*F*(1,36) <1, NS] (Figure 2C). As with silent gap 0–300 ms, results from the silent gap 0–100 ms task reflect an overall impairment of RAP in mutant mice in comparison to wildtype mice, an effect seen regardless of sex. A significant Strain × Gap interaction was also found [*F*(8,288)  = 25.4, *P*<0.001], again indicating that wildtype mice performed better than mutants particularly on the “easier” gap intervals (>50 ms), while both groups had similar difficulties in detecting the shorter gap intervals (<50 ms), thus reducing group differences due to task difficulty. Comparison of mean cued and uncued startle amplitude within the mutant group was again performed to assess silent gap detection at individual gaps. T-tests revealed that mutant mice were capable of cue discrimination at the 75 ms interval only, indicating that mutant mice were generally impaired on this more difficult task, but were still able to perform the task on an easier (longer) condition. Analysis of individual gap detection in wildtype mice found that they were capable of performing silent gap 0–100 ms at all gap intervals down to 2 ms (Figure 2C).

### BXD29-Tlr4^lps−2J^/J mutants show no impairment of sensorimotor ability

**Figure 3:**
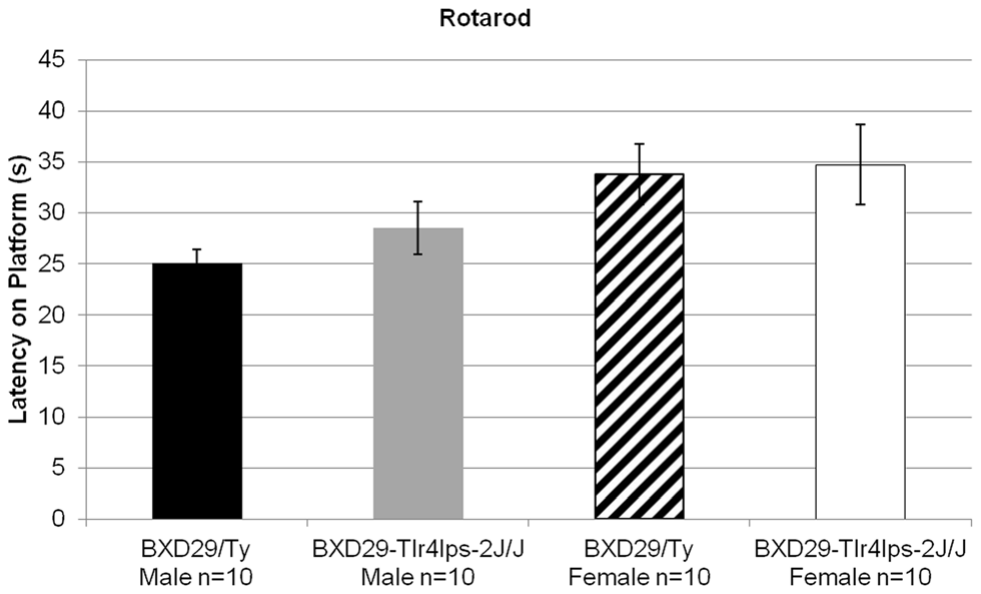
Rotarod performance of BXD29-*Tlr4^lps−2J^*/J mutant and BXD29/Ty wildtype mice. No main effect of Strain or Sex × Strain interaction was observed on the rotarod task, demonstrating the lack of sex differences in the BXD29-*Tlr4^lps−2J^*/J strain of mice.

A univariate ANOVA of rotarod latencies including the between-subjects variables Strain (2 levels: mutant and wildtype) and Sex (2 levels: male and female), was used to assess sensorimotor ability. This analysis revealed no significant differences across Strain [*F*(1,36)< 1, NS] (Figure 3). However, there was a significant main effect of Sex [*F*(1,36)  = 6.8, *P*<0.05], showing that females remained on the rotarod significantly longer than males, regardless of strain. This observation has been reported elsewhere and is consistent with previous findings [42]. The absence of a significant interaction between the variables Strain and Sex [*F*(1,36)<1, NS], coupled with independent samples t-tests, confirmed that there were no strain differences in rotarod ability for either sex.

### Female BXD29-Tlr4^lps−2J^/J mutants show superior Morris water maze ability

Initial assessment of water maze escape latencies using a visual platform control task found no main effects of Strain [*F*(1,36)<1, NS], Sex [*F*(1,36)  = 2.5, NS], or Strain × Sex interaction [*F*(1,36)  = 2.6, NS], providing evidence that all subjects performed comparably in visually locating and swimming to the visible platform. Thus, all subjects were included for further Morris water maze testing.

#### Morris Water Maze (MWM)

**Figure 4:**
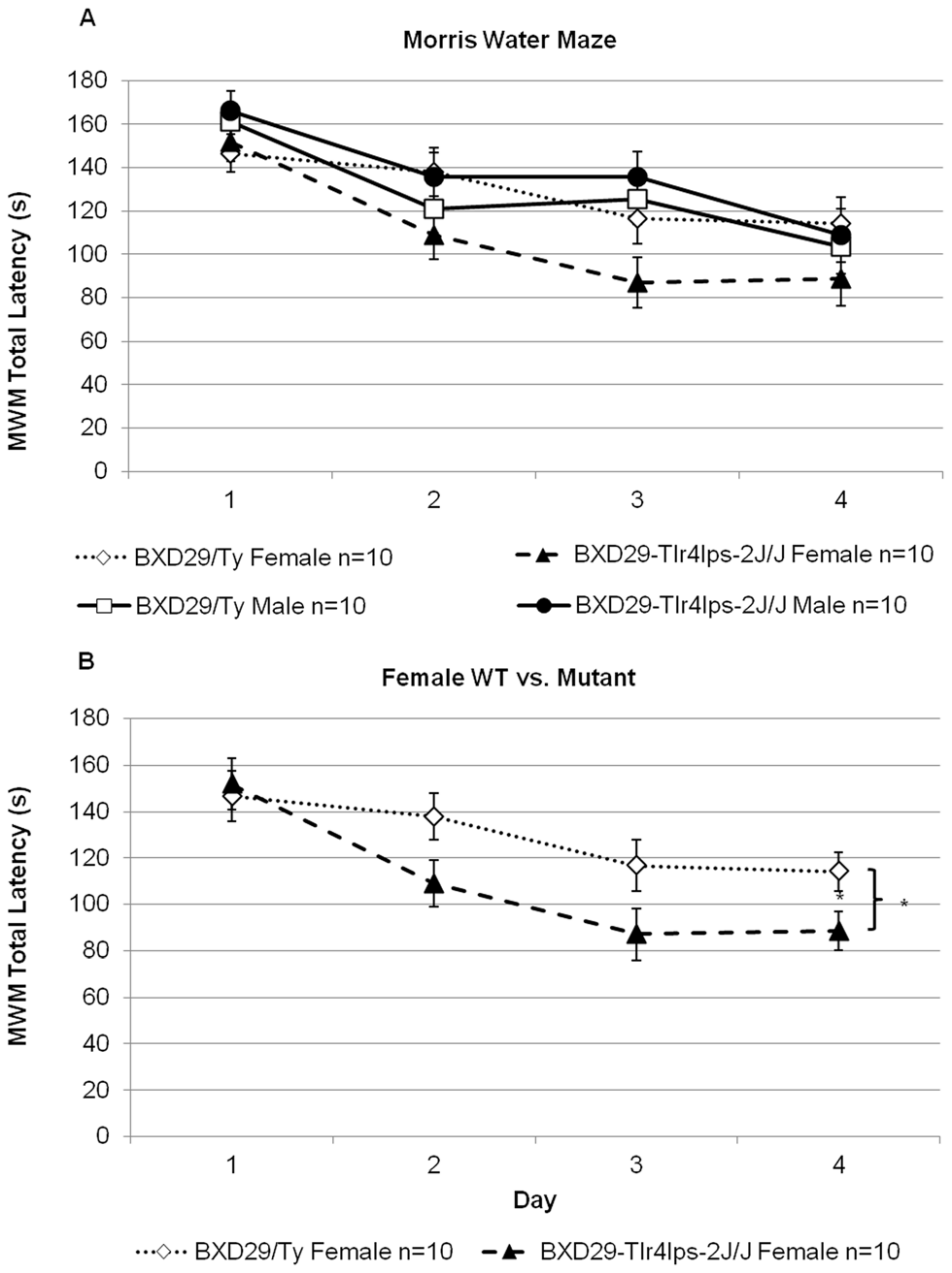
Morris water maze performance of BXD29-*Tlr4^lps−2J^*/J mutant and BXD29/Ty wildtype mice. A) Overall analysis of Morris water maze performance revealed a main effect of Sex and Sex × Strain interaction. B) Further post-hoc analysis indicated that the main effects of Sex and Sex × Strain interaction were pulled by the significantly better Morris water maze performance of female BXD29-*Tlr4^lps−2J^*/J mutant mice in comparison to both male BXD29-*Tlr4^lps−2J^*/J mutant mice and female BXD29/Ty wildtype mice. *P<0.05.

Statistical analysis of MWM escape latencies across four trials using a repeated measures ANOVA with between subject variables of Strain (2 levels: mutant and wildtype) and Sex (2 levels: male and female) and the within subject variable of Day (4 levels), did not reveal an overall effect of Strain [*F*(1,36)<1, NS] (Figure 4A). However, there was a main effect of Sex [*F*(1,36)  = 4.3, *P*<0.05], with females overall taking *less* time to locate the hidden platform. This result runs counter to typical reports wherein female rodents consistently perform worse than males on MWM and related spatial tasks [43]–[45]. This unusual result may be explained by a significant Strain × Sex interaction [*F*(1,36)  = 5.1, *P*<0.05], indicating the sex effect was driven by mutant females (Figure 4B). In fact, further post-hoc analysis using Fisher's least significant difference test found statistically superior performance of the female mutant mice on the Morris water maze as compared to all other groups. This effect was seen when compared not only to the male mutant mice, but also to the wildtype males and females (*P*<0.05). Additional analysis also found a main effect of Day [*F*(1,36)  = 7.16, *P*<0.001], indicating that all subjects improved on the task as testing progressed.

### BXD29-Tlr4^lps−2J^/J mutant mice exhibit enhanced social behaviors

#### Male Vocalizations

**Figure 5:**
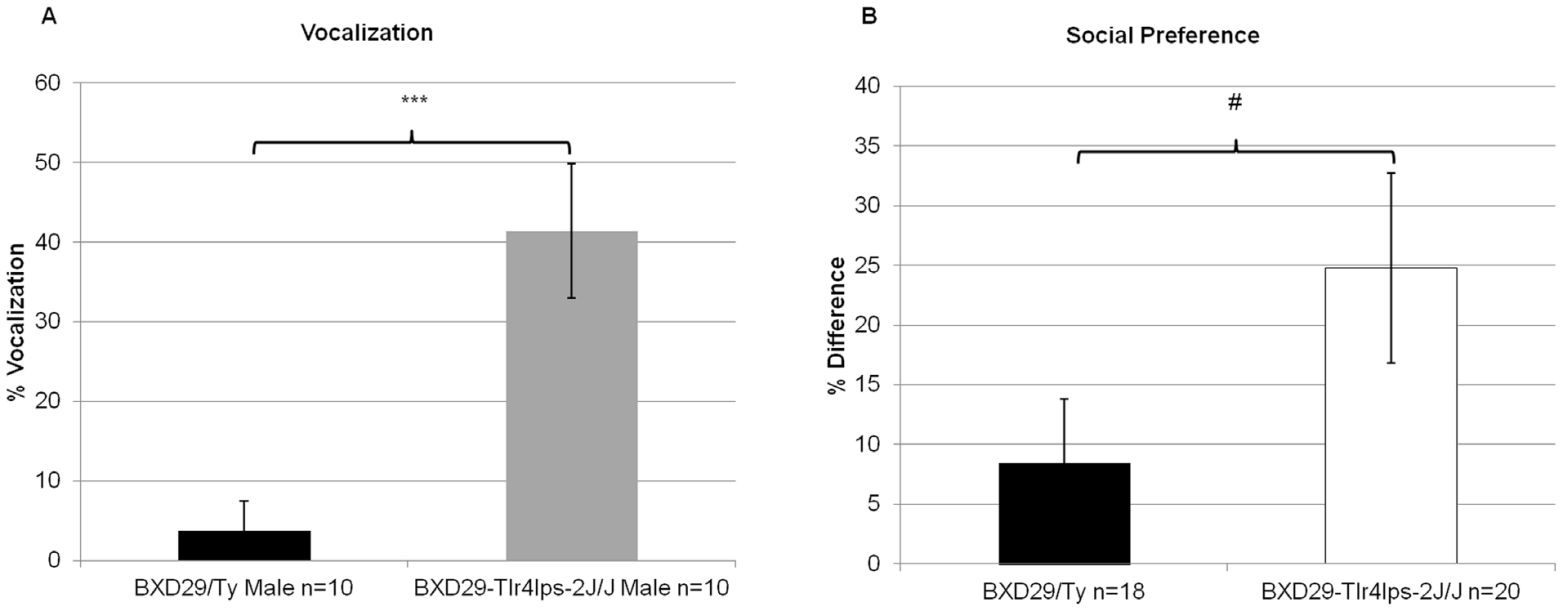
Examination of behavioral response to different social contexts in BXD29-*Tlr4^lps−2J^*/J mutant and BXD29/Ty wildtype mice. A) Analysis of male BXD29-*Tlr4^lps−2J^*/J and BXD29/Ty ultrasonic vocalization emissions when exposed to seven-day-old dirty female bedding demonstrated that male BXD29-*Tlr4^lps−2J^*/J mutant mice spent significantly more time vocalizing in response to dirty female bedding in comparison to male BXD29/Ty wildtype mice. B) Social preference data revealed a nearly significant trend that suggested BXD29-*Tlr4^lps−2J^*/J mutant mice, in general, may prefer increased social interaction with a stranger mouse in comparison to a novel inanimate object. #*P* = 0.1; ***P<0.001.

Examination of male vocalization to estrous female bedding using an independent samples t-test revealed a significant main effect of Strain [*t*(18)  = −4.07, *P*<0.001], indicating that male mutant mice spent significantly *more* time vocalizing when exposed to female bedding in comparison to male wildtype mice (41% vs. 4%; Figure 5A).

#### Social Preference Task

Analysis of social preference data (percent time with stranger mouse) using a univariate ANOVA with between subject variables Strain (2 levels: mutant and wildtype) and Sex (2 levels: male and female) suggests a trend toward a significant main effect of Strain [*F*(1,34)  = 2.6, P = 0.1] (Figure 5B). Although no definitive conclusions can be drawn from this result due to a high level of variability, the trend shows that mutant mice spent a greater percent of time with the stranger mouse than the novel toy in comparison to wildtype mice. In fact, these findings strongly suggest *increased* sociability in the mutant mice based on the fact that social preference scores were more than doubled in mutant mice. Finally, there was no main effect of Sex [*F*(1,34)<1, NS] or Strain × Sex interaction [*F*(1,34)<1, NS], indicating that this enhanced social behavior was seen for both male and female mutants.

## Discussion

Neuronal migration disorders clinically show a wide range of neuroanatomical and behavioral manifestations, with deficits ranging from mild to severe. Moreover, behavioral anomalies are seen across cognitive, social, and neurophysiological domains. Although the pattern and severity of behavioral and cognitive outcomes seem to vary depending on extent and localization of the malformation(s), a precise diagnostic prediction of symptoms associated with specific anomalies has not been possible, and this issue requires additional study (see [7], for review). Furthermore, clinical data show a greater prevalence of males diagnosed with neurodevelopmental disorders associated with neuroanatomical anomalies (such as autism and developmental dyslexia), and males also tend to present with more severe behavioral outcomes related to language and reading disability (see [27] for review). To better examine the relationship between severe neuroanatomical malformations, as well as the potential role of sex in the manifestation of behavioral abnormalities associated with language-related dysfunction and social context, male and female BXD29-*Tlr4^lps−2J^*/J mouse mutants (and their coisogenic and sex matched controls) were examined using a battery of assessments intended to model core non-verbal aspects of language related dysfunction, sensorimotor, and social ability.

### Deficits in rapid auditory processing behavior in BXD29-Tlr4^lps−2J^/J mice, but no sex differences

Within the current study, we were able to replicate and expand upon the behavioral findings initially described in Rosen et al. 2013. Specifically, the BXD29-*Tlr4^lps−2J^*/J mutant mouse strain again displayed severe impairments in RAP despite typical hearing and PPI ability. Although the wildtype BXD29/Ty mice were capable of gap detection up to 2 ms in duration, mutant mice were only able to discriminate silent gaps longer than 75 ms in duration. In addition, despite significant gap detection at the longer intervals, the mutant mice were unable to attain the same level of discrimination as wildtype mice even at longer durations.

Interestingly, both male and female mutant mice were comparably impaired in silent gap detection in comparison to their respective coisogenic controls, indicating no sex difference in RAP ability. This result deviates from findings associated with previous research where sex differences in RAP ability favoring females *were* seen in other rodent models of neuroanatomical malformation and cortical injury [17], [23], [46], [47]. However, it must be noted that these studies which reported a female “advantage” in outcomes following disruptions in cortical development all utilized models of **injury** induced damage and disruption [23], [47]. Although the mechanisms of altered cortical development and subsequent reorganization leading to RAP impairment remain unclear, studies of injury-based models examining disrupted cortical development have also shown sex-dependent mechanisms associated with apoptosis, as well as data implicating a role for estrogen modulation in neuroprotective pathways [48], [49]. Furthermore, subsequent research following the discovery of sex differences in outcomes using the induced focal microgyria model [17] found that the presence of circulating androgens in males and females provided exogenously following injury resulted in behavioral deficits in treated females [47] and also aberrant reorganization of the medial geniculate nucleus (MGN; [50]). That is, females with induced microgyria (not exposed to androgens) displayed comparable MGN morphology to unaffected females, thus suggesting that sex differences observed in impaired RAP ability and thalamic anomalies could be explained in part by early androgen exposure concurrent to cortical injury. Evidence from injury based models, including focal microgyria and neonatal hypoxia-ischemia models, thus suggest that sex-dependent mechanisms (which may in turn reflect modulating effects of androgens and/or estrogens) associated with apoptosis and cellular reorganization could contribute to the female advantage observed in subsequent RAP ability. In contrast to results from these injury models, cortical abnormality in the BXD29-*Tlr4^lps−2J^*/J mutant in the current study was *not* a result of an experimentally induced injury that could initiate a sexually dimorphic apoptotic cascade. Therefore, the *lack* of sex differences in RAP ability in mutant mice suggests that gonadal steroids do *not* mediate mechanisms associated with severe RAP impairment when that altered behavior is genetically mediated. These results are consistent with a recent study conducted by Szalkowski et al. 2013, which examined behavioral sex differences following genetic knockdown (RNAi) of the rodent homolog for the dyslexia candidate risk gene, DYX1C1 (Dyx1c1 in rodents) [51]. This study reported no sparing of female performance in RAP behavior, with Dyx1c1 (RNAi) females showing deficits comparable to those seen in treated males. Taken together with evidence from injury based models, findings suggest that cortical disruption as a result of non-injury related genetic factors may not necessarily elicit a female advantage, even though such an advantage is often seen in outcomes following injury-induced developmental cortical disruption [17], [23], [47]. As a caveat, it must be noted that Peiffer et al. 2002, demonstrated a female advantage in RAP among a subset of male and female BXSB mice expressing Layer 1 ectopias. Findings from this study are difficult to interpret in the context of sex interactions with injury versus genetics, specifically because it is not known what factors lead some BXSB mice and not others to express ectopias [52].

### Superior spatial water maze learning in female BXD29-Tlr4^lps−2J^/J mutant mice

Superior Morris water maze performance observed in female BXD29-*Tlr4^lps−2J^*/J mutant subjects was an unexpected finding in the current study, especially since prior studies with male mutant mice revealed comparable Morris water maze learning to male wildtype mice. Moreover, this finding was intriguing because male rodents tend to perform better than females on the Morris water maze as a baseline (see [53], for review). However, there have been a few instances in the rodent literature reporting a female superiority in spatial learning ability using the Morris water maze [52]. For example, this sex-reversal was seen in BXSB mice, an autoimmune strain of mice known to have spontaneously occurring molecular layer neural ectopias in a subset of the population. In the study conducted by Schrott et al. 1993, female BXSB mice swam significantly faster to the hidden platform, while traveling a shorter distance to locate the escape platform [50]. Although the results from Schrott et al. 1993 seem to correspond to the current findings associated with a female advantage in spatial learning in models of neuronal migration anomalies, it must be noted that the presence of neural ectopias in BXSB mice are not 100% penetrant in comparison to malformations observed in BXD29-*Tlr4^lps−2J^*/J mice. Additionally, there was no analysis comparing ectopic versus non-ectopic female performance in this sample, thus it remains unclear whether the presence of a neuroanatomical anomaly in BXSB females could have contributed to better Morris water maze performance. Despite previous research alluding to a potential female advantage in spatial learning and memory ability associated with neuronal migration anomalies, the novel finding of a female superiority associated with the Morris water maze task in BXD29-*Tlr4^lps−2J^*/J mutant mice must be examined further to verify the results. An additional possibility is that enhancements are sometimes associated with deficits, and the Morris water maze “enhancement” may have only been evidenced in females due to their lower initial baseline performance.

### Hyper-sociability in BXD29-Tlr4^lps−2J^/J mutant mice

Within a social context, male mice may produce ultrasonic vocalizations when they encounter a female mouse (particularly in estrus), or are exposed to female urinary pheromones [34], [54]–[56]. We found that when male BXD29-*Tlr4^lps−2J^*/J mutant and BXD29/Ty wildtype mice were exposed to dirty female bedding (bedding containing estrus female urine), mutant mice spent a substantially greater amount of time emitting ultrasonic vocalizations as compared to wildtype mice (41% vs. 4% of session). Additionally, comparison of social preference to a stranger mouse versus a novel inanimate toy found a trend that suggested mutant mice, regardless of sex, preferred *more* social interaction with the stranger mouse in comparison to wildtype mice (8% social preference in wildtype, 25% preference in mutants). Together these two findings suggest that BXD29-*Tlr4^lps−2J^*/J mutants exhibit hyper-social behaviors in comparison to BXD29/Ty wildtype mice.

Previous work examining normal male mouse ultrasonic vocalization behavior to female urine found that sexually naïve males (as investigated in the current paradigm) do produce vocalizations to concentrated amounts of freshly voided female urine (in the absence of a female). However, sexually experienced males (provided female interaction three minutes a day for eight days) vocalized significantly more when exposed to the same stimulus [34]. Furthermore, it was found that both sexually naïve and experienced males vocalized less when exposed to concentrations of older urine (1–13 hours), and that vocalization behavior decreased over time when exposed to female urine without the presence of a female mouse [34]. These findings suggest that results could also reflect differences in “social acuity” regarding whether a fertile female may actually be present. Specifically, wildtype male BXD29/Ty subjects exhibited minimal vocalization behaviors (<4% of total session), and this might be expected since female urine concentration was more diffuse within the dirty female cage bedding and most likely contained a higher concentration of older urine (>1 hour) in comparison to freshly voided. However, when exposed to identical female dirty bedding, mutant BXD29-*Tlr4^lps−2J^*/J mice emitted ultrasonic vocalizations for more than 40% of the total test session. This indicated that mutant mice were overtly sensitive to the dirty bedding stimulus, which resulted in a production level of vocal social behaviors more typical of that seen when a female is either physically present, or thought to be nearby [34], [54]–[56]. Thus, one interpretation could be that mutant males have impairments in processing pheromonal (social) information in terms of relevance. On the other hand, our assessments of social preference also suggest that mutant mice are more social than wildtype and prefer social interaction with another mouse in comparison to a novel inanimate object at higher than normal levels. Specifically, mutant mice spent 25% more time with the same sex stranger mouse versus the toy in comparison to 8% more time observed in wildtype mice. Therefore, data collectively from the two paradigms assessing behavioral response to different social contexts suggest that mutant BXD29-*Tlr4^lps−2J^*/J mice exhibit more hyper-social behaviors in comparison to BXD29/Ty wildtype controls.

### BXD29-Tlr4^lps−2J^/J mutant mice: a neuroanatomical model for neurodevelopmental disorders?

There are numerous molecular pathways that regulate neuronal migration (see [57], for review). Several of these gene networks – identified through association studies using different types of gene variants including single nucleotide polymorphisms and copy number variants – have been implicated in complex neurodevelopmental disorders ranging from autism, schizophrenia, and developmental dyslexia [58]–[60]. Unfortunately, the molecular etiology of the aberrant neuroanatomical phenotype observed in BXD29-*Tlr4^lps−2J^*/J mutant mice remains unknown. However, previous genetic backcross experiments observing the pattern of inheritance for the cortical malformations do suggest that the phenotype is mediated by the expression of two independent autosomal recessive genes, but additional sequencing experiments comparing mutant and wildtype mice must be conducted in order to further isolate and identify the responsible genes [29]. It must be noted that the *Tlr4* mutation identified by Cook and colleagues was determined to not be causal in the development of the cortical mutations [29], [31].

Despite the lack of clear molecular insights associated with the BXD29-*Tlr4^lps−2J^*/J model, the complete neuroanatomical phenotype of BXD29-*Tlr4^lps−2J^*/J mice, including partial callosal agenesis and subcortical nodular heterotopia bilaterally located near the midline between the retrosplenial cortex and parietal and visual cortices do share overlapping (though not identical) characteristics of other malformation phenotypes commonly observed in human disorders of neuronal migration. For example, the subcortical location and rather diffuse nature of the malformation is similar in respects to subcortical band heterotopia [7], [29], [61]. However, the nodular structure of the BXD29-*Tlr4^lps−2J^*/J malformation is not analogous to subcortical band heterotopia, and is more reminiscent of periventricular nodular heterotopia – a malformation located along the ventricles [7], [29]. The presence of periventricular nodular heterotopia has been indentified in patients with both dyslexia and Williams syndrome [3], [62] – disorders that are associated with impairments in reading ability and excessive sociability, respectively [39], [63]. Behaviors associated with both dyslexia *and* Williams syndrome have been modeled in the current study using RAP and sociability paradigms, respectively. Importantly, we found altered behavior associated with the BXD29-*Tlr4^lps−2J^*/J mutant mice within both of those behavioral domains.

Similarly, callosal agenesis in the human population is often also observed with other malformations of cortical development, and is associated with a wide range of aberrant behaviors across cognitive and social domains [40], [41]. For example, atypical callosal morphology featuring a shorter and thinner corpus callosum is present in a subset of patients with Williams syndrome [64]. However, notably, our pattern of findings do not provide an ideal match for Williams syndrome, since this population presents with an atypical presentation of language (though reading impairments are in fact associated with intellectual disability in this population [65]). Nonetheless, the current mouse model could provide a platform to trace associations between specific neural anomalies and functional anomalies relevant to a range of disorders.

## Conclusion

We report novel findings of rapid auditory processing deficits in both male and female mutant BXD29-*Tlr4^lps−2J^*/J mice. We also present evidence for enhanced female BXD29-*Tlr4^lps−2J^*/J performance in Morris water maze learning, as well as hyper-sociability in mutant mice, regardless of sex. In closing, the BXD29-*Tlr4^lps−2J^*/J mouse is not a perfect model for a singular neurobehavioral disorder, nor does it fit within a single phenotype of known human neuronal migration disorders. However, various components of the behavioral and neuroanatomical phenotype of BXD29-*Tlr4^lps−2J^*/J mice share commonalities that can be translated to different types of neuronal migration and neurobehavioral disorders, allowing for a general examination of the relationship between neuronal migration malformation and behavior. Moreover, the lack of sex differences observed in RAP behavior within the mutant group do not directly address the lower incidence ratios favoring females over males in the diagnosis of dyslexia, SLI, and autism (2∶1, 3∶1, and 4∶1, [27], [66], [67]). As such, the question remains why more males are diagnosed with language related neurodevelopmental disorders, many of which have a strong genetic component. For example, it may be that other environmental risk factors that exacerbate or interact with genetic risk (i.e., perinatal fevers, teratogens) may be the locus of female “advantage” as seen in rodent models of developmental injury and cortical disruption [17], [23], [47]. Questions regarding the associations between genetic and environmental risk, sex, anomalies in cortical development, and subsequent impairments in behavior and outcomes remain a critical ongoing issue in neurodevelopment research. Importantly, our findings suggest that the BXD29-*Tlr4^lps−2J^*/J mutant model may provide an ideal platform for continuing to tease apart these issues in future studies. As such, additional research using the BXD29-*Tlr4^lps−2J^*/J strain should be undertaken to examine the relationship between neuronal migration anomalies, neural connectivity, behavior, and the role of sex in the manifestation of clinical outcome. The insights provided by these future studies could provide crucial clues to develop a neuroanatomical and reorganizational neurocircuitry that may explain how aberrant neuronal migration insult can lead to impairments within specific behavioral domains.
